# Associations between an inflammatory diet index and nonunion: a prospective study of 172,839 UK biobank participants

**DOI:** 10.3389/fnut.2025.1640259

**Published:** 2025-07-14

**Authors:** Jiemao Su, Wenxuan Fan, Keyu Kong, Yifan Wang, Zanjing Zhai, Jingwei Zhang, Minghao Jin, Yansong Qi, Yongsheng Xu

**Affiliations:** ^1^Orthopedic Center (Sports Medicine Center), Inner Mongolia Autonomous Region People's Hospital, Hohhot, China; ^2^Shanghai Key Laboratory of Orthopaedic Implants, Department of Orthopaedic Surgery Shanghai Ninth People’s Hospital, Shanghai Jiaotong University School of Medicine, Shanghai, China

**Keywords:** inflammation, diet, nonunion, prospective studies, incidence

## Abstract

**Purposes:**

This study utilizes prospective cohort data from the UK Biobank to investigate the association between the energy-adjusted dietary inflammation index (E-DII) and the development of fracture nonunion.

**Methods:**

In this study, COX regression was used to analyze the correlation between E-DII and nonunion. Among 172,839 participants free of prior nonunion at baseline, 2,341 (1.4%) developed nonunion during a median follow-up of 14.2 years. E-DII scores, calculated from five separate 24-h dietary recall assessments, were used to quantify dietary inflammatory potential, with higher values indicating pro-inflammatory patterns.

**Results:**

Multivariable-adjusted analyses revealed that participants with anti-inflammatory dietary patterns (E-DII < −1) exhibited a significantly elevated risk of impaired fracture healing compared to those with pro-inflammatory diets (E-DII > 1), yielding an adjusted hazard ratio (HR) of 1.89 (95% CI: 1.45–3.11). A nonlinear U-shaped dose–response relationship was identified, with the nadir of nonunion risk observed at E-DII values between 0.3 and 1.2. Conversely, values outside this range were associated with progressively higher risks. Transcriptomic profiling identified differential expression of 35 inflammation-related genes—including CD3E and CX3CR1—significantly downregulated in nonunion cases compared to controls. These genes are functionally enriched in pathways governing immune response regulation and leukocyte activation.

**Conclusion:**

These findings propose that a moderately pro-inflammatory dietary pattern may confer protection against impaired bone healing, whereas both strongly anti-inflammatory and excessively pro-inflammatory diets were associated with compromised healing outcomes. Based on these results, tailored dietary strategies designed to optimize inflammatory homeostasis during fracture recovery are recommended to enhance clinical outcomes.

## 1 Introduction

Bone is an exceptional biological material distinguished by its capacity for complete regeneration under optimal healing conditions. Despite this inherent regenerative potential, a notable proportion (10–15%) of fracture cases exhibit compromised healing processes, resulting in either delayed union or non-union outcomes (1, 2). The estimated annual incidence rate of nonunion fractures in the UK is 0.02% within the general population, with the highest prevalence noted among young and middle-aged males (3). The clinical implications of long bone nonunion pathology are complex and multifaceted, including persistent nociceptive symptoms, substantial functional limitations, and associated psychological morbidity (4). The impact of bone nonunion is substantial, placing a considerable financial strain on patients’ families and contributing to a decline in social productivity. According to reports, each case of bone nonunion incurs an average societal cost of approximately 79,000 pounds, which poses a considerable obstacle to social and economic progress (5, 6).

Under normal physiological conditions, the fracture healing process typically progresses through three interconnected stages: the inflammatory response phase, the fracture repair phase, and the callus remodeling phase (7, 8). These stages are not entirely distinct but exhibit significant overlap and interdependence. The entire fracture healing process generally takes approximately 2 years to complete. Notably, the inflammatory response exerts the most pervasive influence. This phase is initiated by the release of inflammatory mediators from damaged blood vessels following the fracture, leading to the development of a hematoma enriched with inflammatory cells and cytokines. This process not only facilitates local inflammation but also sets the stage for subsequent stages of fracture healing (8, 9). The formation of callus is influenced by a multitude of factors, including anatomical structure and biomechanical properties (8, 9). For instance, in unstable fractures, bone healing typically proceeds through endochondral ossification. The process unfolds as follows: cartilage tissue initially forms at the fracture site, followed by gradual differentiation and eventual transformation into bone tissue (10). During the chondrogenesis stage, chondrocytes first proliferate in large numbers, and then further hypertrophy and undergo metaplasia. Through these processes, the cartilage tissue in the fracture gap gradually forms bone tissue, creating a bony callus and stimulating the development of new blood vessels (8, 11). The newly formed blood vessels in the fracture area are rich in mesenchymal stem cells (MSCs) and inflammatory cells, including monocytes. These recruited cells gradually differentiate into osteoclasts and osteoblasts, which promote fracture healing and callus remodeling (12).

During the bone remodeling phase, the coupling between osteoclasts and osteoblasts plays a critical role. Osteoclasts degrade bone through the polarized secretion of proteolytic enzymes, such as cathepsin K, and acid. These agents, respectively, hydrolyze the organic matrix and dissolve the mineral components of bone. Proton and enzyme secretion is directed into the resorption lacuna. This compartment is sealed off from the surrounding bone microenvironment by the dense, actin-rich podosome belt encircling the osteoclast’s ruffled border, forming the sealing zone (13, 14). Following the bone resorption phase, the coupling mechanism facilitates the recruitment and differentiation of mesenchymal-derived osteoprogenitor cells within the resorption lacunae. Upon maturation into osteoblasts, these cells align along the eroded bone surface and secrete the organic component of bone, termed osteoid. This osteoid subsequently undergoes mineralization over time through the deposition of hydroxyapatite crystals (15). During osteoid secretion by osteoblasts, a subset of these cells becomes embedded within the matrix and ultimately differentiates into osteocytes. Disruption of this coupling mechanism disturbs the delicate physiological balance between resorption and formation, thereby underlying various skeletal pathologies (16). Existing research has demonstrated that osteoclast number exerts a more profound influence on the callus remodeling phase. Elevated osteoclast numbers lead to a significant quantitative reduction in both the number and thickness of newly formed trabecular bone (17).

The established association between dysregulated bone remodeling, inflammation, and skeletal pathology underscores the significance of systemic inflammation as a modulator of physiological processes. Chronic low-grade systemic inflammation represents a well-established mediator influencing not only bone metabolism but also a wide range of other chronic diseases.

During the inflammatory phase of fracture healing, a large number of immune cells are transported via the bloodstream to the fracture site, where they accumulate and release pro-inflammatory cytokines. These cytokines stimulate angiogenesis, cartilage formation, and promote the differentiation of fibroblasts and osteoblasts (18). Suppressing inflammation during this phase prevents the necessary inflammatory signals from gathering at the fracture site, thereby hindering the early formation of essential components such as blood vessels and ultimately leading to impaired healing (19). In the bone remodeling phase, osteoblasts and osteoclasts play a central role, and this stage is also regulated by pro-inflammatory cytokines, such as IL-1 and IL-6 (20). Inhibiting inflammation at this stage can disrupt the remodeling process and prevent the restoration of normal bone structure.

A substantial body of evidence demonstrates that dietary patterns significantly influence the regulation of chronic inflammation, thereby affecting the development and progression of cardiovascular diseases, type 2 diabetes, and specific cancers (21, 22). Current research demonstrates that the Dietary Inflammatory Index (DII) score is significantly associated with changes in systemic inflammatory markers, particularly serum levels of C-reactive protein (CRP) (23). It is known to all that CRP is widely recognized as one of the most sensitive biomarkers for detecting inflammatory diseases, thereby indirectly supporting the notion that DII can influence the occurrence and progression of these conditions. Initially developed in 2009, DII serves as a comprehensive scoring system to evaluate dietary patterns by ranking individual foods along a range from anti-inflammatory to pro-inflammatory. Compared with other dietary scoring systems, DII offers significant practical advantages in terms of ease of use (24). Compared to other diet-quality scores or indices, the DII specifically addresses the inflammatory effects of foods and nutrients, offering a more precise approach to understanding how diet influences inflammation and health (25–28). Moreover, the DII has been validated through measurements of circulating CRP levels and other inflammatory biomarkers, thereby establishing its reliability as an indicator of dietary effects on systemic inflammation (24, 29, 30). Over the past ten years, over 750 studies have utilized the DII to examine its associations with morbidity and mortality, thereby reinforcing its significance in both clinical and public health research (31–34).

Research on the Dietary Inflammatory Index (DII) indicates that individuals with elevated DII scores typically exhibit reduced dietary vitamin D intake (35). Furthermore, lower DII scores correlate with diminished systemic levels of inflammatory biomarkers compared to pro-inflammatory dietary patterns, highlighting the critical role of dietary composition in modulating inflammatory pathways (35). Accumulating evidence underscores associations between pro-inflammatory diets (higher DII scores) and adverse health outcomes, including greater severity of non-alcoholic fatty liver disease (NAFLD) (36), heightened COVID-19 morbidity (37), and elevated all-cause mortality risk (38). In orthopedics, studies suggest that anti-inflammatory dietary interventions may attenuate sarcopenia progression by suppressing chronic inflammation and mitigating oxidative stress (39).

Bone nonunion is a complex condition resulting from the interplay of multiple factors. Therefore, implementing appropriate preventive measures before nonunion occurs represents the most cost-effective approach to treatment. Given that dietary patterns significantly influence systemic inflammation, they provide an important avenue for investigating the prevention of bone nonunion. Existing research has demonstrated a link between dietary habits and the risk of nonunion, highlighting the potential significance of dietary factors in preventive and interventional strategies (40–42). Although the above-mentioned studies have demonstrated the correlation between nonunion of bones and inflammation as well as diet, they are not sufficient to prove that dietary structure can affect nonunion of fractures (42). Existing research evidence indicates that diet can influence fracture healing through multiple pathways such as reducing inflammation levels, decreasing oxidative stress, and enhancing the body’s immune system (43).

**Figure 1:**
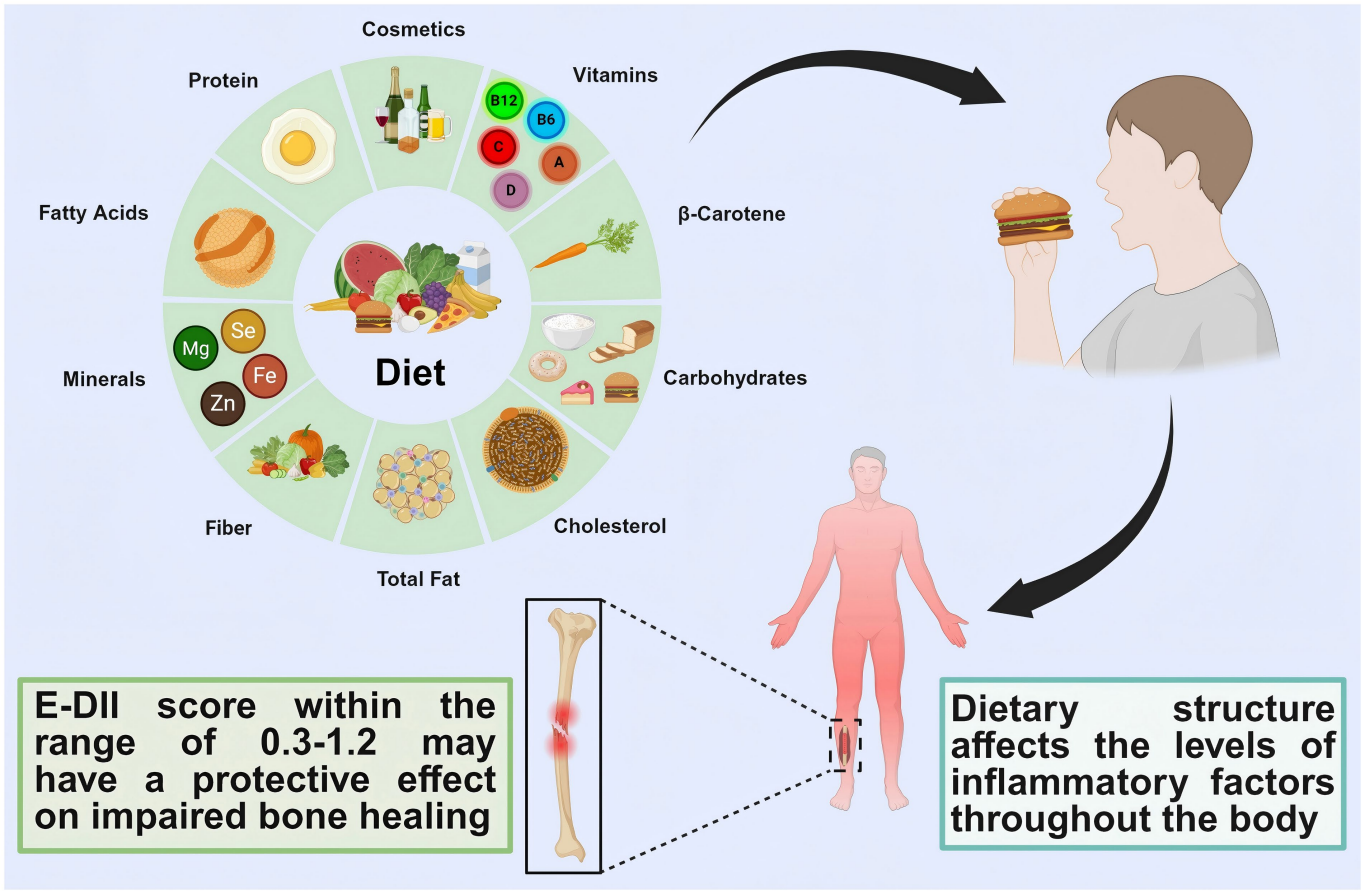
Graphic summary.

This study aims to explore the correlation between dietary structure and bone nonunion by synthesizing findings from prior research. Furthermore, a prospective cohort study leveraging the UK Biobank database will be implemented to generate higher-precision scientific evidence. We hypothesize that a moderate pro-inflammatory dietary potential is inversely associated with the risk of fracture nonunion, whereas both high pro-inflammatory and high anti-inflammatory dietary potentials are positively associated with increased nonunion risk (Figure 1).

## 2 Methods

The UK Biobank is a large-scale longitudinal study involving over 500,000 individuals aged 37–73 years at baseline, accounting for 5.5% of the invited population (44). Between 2006 and 2010, volunteers visited 22 designated assessment centres located in Scotland, England, and Wales (45, 46). During these sessions, participants engaged in touch-screen-based surveys, received standardized health evaluations, and donated biological specimens (blood, urine, and saliva). Additional information about the study’s methodology can be found on the UK Biobank website at http://www.ukbiobank.ac.uk.

### 2.1 Ethical approval

All participants provided written informed consent prior to data collection in the UK Biobank, which received ethical approval from the North West Multi-Centre Research Ethics Committee (REC reference: 11/NW/0382). All experiments conducted in this study strictly adhered to the relevant guidelines and regulations, with no instances of violation. Access to the database and execution of this study were authorized under UK Biobank Application Number 93966.

### 2.2 Diet Inflammatory Index

Participants used the Oxford WebQ, an online platform, to complete their 24-h dietary assessment by reporting the types and amounts of food and drinks they consumed during the prior day (47). Researchers queried participants about their dietary intake to gather detailed information on their eating habits. Each participant completed the assessment five times at different intervals, and the average of these assessments was used for analysis (48). Data collection occurred over a three-year period from April 2009 to June 2012. For more detailed information, please refer to the 24-h recall category (Category ID: 100090).

This study incorporated participants who completed at least one dietary assessment questionnaire. The Dietary Inflammatory Index (DII) score for each individual was derived from mean intake values across multiple dietary evaluations. Originally developed by Shivappa et al. and comprising 45 dietary parameters (29). The DII employs globally standardized reference values established through a systematic review of ~2000 studies linking dietary components to inflammatory biomarkers, supplemented by weighted scoring of 11 international dietary datasets (49). For this analysis, we aligned the 45 original DII parameters with 24-h dietary recall data from the UK Biobank, yielding 27 evaluable components (50): alcohol, vitamins (B12, B6, A, C, D, E), beta-carotene, carbohydrates, cholesterol, total fat, fiber, minerals (Fe, Mg, Se, Zn), fatty acids (MUFA, omega-3, omega-6, SFA, trans-fatty acids), protein, riboflavin, niacin, thiamin, tea consumption, and total energy intake. Individual DII scores were computed by multiplying each component’s inflammatory effect weight by its daily intake. To account for energy intake variability, scores were normalized to a 1,000 kcal/day standard, generating the energy-adjusted DII (E-DII) (30). Full methodological details for E-DII calculation are available in prior publications (30, 35, 51): anti-inflammatory (E-DII < -1), neutral (E-DII ≥ -1 to≤1), and pro-inflammatory (E-DII > 1) (31).

### 2.3 Nonunion

Nonunion fracture diagnoses were classified according to the International Classification of Diseases, Tenth Revision (ICD-10) codes M84.0–M84.4, M84.8, and M84.9, sourced from the UK Biobank (Data Category 1712). These codes identify delayed or impaired fracture healing and related musculoskeletal complications. For comprehensive code definitions and diagnostic criteria, refer to the UK Biobank resource: https://biobank.ndph.ox.ac.uk/showcase/label.cgi?id=1712.

Nonunion was defined as hospitalization resulting from nonunion and identified through linked hospital records. The dates and sources of diagnosis were obtained from the diagnosis certificates maintained by the NHS Information Centre for England and Wales, and the NHS Central Register for Scotland (35). The cut-off date for this study is set as August 2023. Any data points occurring after this date are censored and recorded as of August 2023.

### 2.4 Covariates

Baseline age was determined using the participants’ dates of birth and their initial assessment data. Sex was self-reported by participants at baseline. Socioeconomic status, specifically measured as deprivation, was assessed using the Townsend score derived from the residential postcodes (52). Ethnicity was self-reported and classified into two categories: White and Other. Smoking status, as reported by participants, was categorized into three groups: never smoked, former smoker, and current smoker. The use of medications for chronic conditions, including those for cholesterol, blood pressure, and diabetes, was determined using baseline data. Sleep duration and morning wake time were included as covariates in the analysis. Educational attainment was also considered, with individuals holding a college degree or higher classified as having advanced education, those with secondary school or equivalent classified as having primary education, and all others classified as having primary education. Further details on the measurement methods can be found on the UK Biobank website.^1^

### 2.5 Expression of inflammatory related differential genes in patients with bone nonunion

To further investigate the association between inflammatory diet and fracture nonunion, we explored potential target genes using non-UK Biobank datasets. Given the current lack of datasets specifically linking dietary inflammation to nonunion, this study utilized datasets associated with both inflammation and bone nonunion for target gene screening. This approach is theoretically justified, as the E-DII ultimately influences the development of nonunion through the modulation of serum inflammatory factor levels. This search yielded the peripheral blood gene expression dataset GSE93138, profiled on the GPL6244 platform. The dataset comprises samples from 8 patients with acute injury fracture nonunion (<7 days after injury) and 4 healthy volunteers serving as controls (all participants were over 18 years old). Using the keywords “nonunion,” “bone fractures,” and “*homo sapiens*,” we queried the GEO public database (accession: GSE93138; https://www.ncbi.nlm.nih.gov/geo/) to identify gene expression profiles relevant to bone nonunion (53). The genes associated with inflammation were obtained from the Genecard database, which contains 12,365 genes. Use the “GEO2R” of GEO to screen the differentially expressed genes between bone nonunion samples and healthy samples (|log FC| < −1.2 and *p* < 0.05 after correction as the threshold). We intermixed the screened differentially expressed genes with inflammation-related genes. Finally, we obtained the expression of inflammation-related differentially expressed genes in patients with bone nonunion and enriched the inflammatory differential genes by KEGG and GO.

### 2.6 Statistical analyses

This study employed the Cox proportional hazards model to assess the correlation between E-DII and nonunion of bones. The scores calculated were classified using the method mentioned in the previous section. The pro-inflammatory group was set as the control group in this study, and the results were presented in the form of hazard ratios (HR). The time experienced in this study was recognized as a time-dependent covariate and was involved in the analysis of the results.

**Figure 2:**
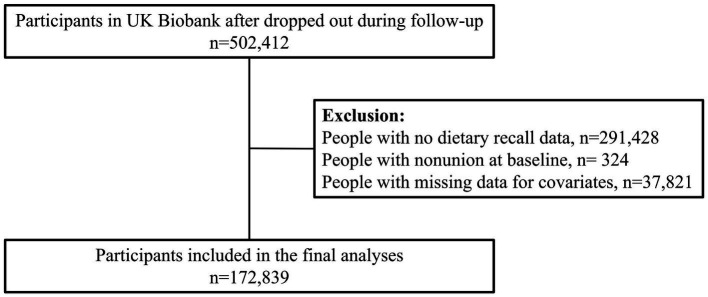
Diagram of participants included in the analyses. Created with BioRender.com.

In the research model, we utilized penalized spline curves to evaluate the association between the E-DII score and bone nonunion. In the penalized spline analysis, the pro-inflammatory group served as the reference group (54). The proportional hazards assumption was assessed using Schoenfeld residuals, in accordance with the guidelines specified by the expert panel. We excluded patients who had nonunion at or before baseline (*n* = 324) (Figure 2), thereby significantly reducing the risk of reverse causality. This study designed the following four models for the covariates mentioned above. Model 0 only analyzed the correlation between E-DII and nonunion of bones. Model 1 included age, gender, race and economic status. Model 2 newly introduced health-related variables. Model 3 further added lifestyle-related factors (55–58).

Calculated E-DII scores were presented as means with standard deviations (SD), and categorical variables were reported as frequencies and percentages (%). All data analyses in this study were conducted using R version 4.2.2, and a two-tailed *p*-value < 0.05 was considered statistically significant.

## 3 Result

Following the exclusion of individuals with prevalent nonunion at baseline or incomplete covariate information, 172,839 participants were retained for analysis (Figure 2). Over a median follow-up of 14.2 years (IQR: 13.5–14.8), nonunion events occurred in 2,341 participants (1.4%). Baseline characteristics stratified by energy-adjusted Dietary Inflammatory Index (E-DII) are presented in Table 1. Participants with E-DII scores <1 had a higher mean age than the overall study population and were predominantly female. Furthermore, 87.19% of these participants were postmenopausal women (defined as menopause occurring at >45 years of age).

**Table 1:** Descriptive characteristics by E-DII categories are presented as means with SD for quantitative variables and as frequencies and percentages for categorical variables.

Characteristics	Total	Anti-inflammatory	Neutral	Pro-inflammatory
*n* (%)	172,839	1,129 (0.65)	85,443 (49.44)	86,267 (49,91)
Baseline age (years), mean (SD)	55.8 (8.0)	56.0 (7.8)	56.6 (7.7)	55.1 (8.2)
Sex, *n* (%)				
Woman	91,809 (53.1)	886 (78.5)	51,545 (60.3)	39,378 (45.6)
Man	81,030 (46.9)	242 (21.5)	33,898 (39.7)	46,889 (54.4)
Deprivation index, mean (SD)	−1.60 (2.86)	−1.19 (3.10)	−1.70 (2.81)	−1.52 (2.90)
Ethnicity, *n* (%)				
White	156,805 (90.7)	997 (88.3)	77,591 (90.8)	78,237 (90.7)
Other	16,034 (9.3)	132 (11.7)	7,852 (9.2)	8,030 (9.3)
Smoking status, *n* (%)				
Never	97,577 (56.5)	661 (58.5)	49,137 (57.5)	47,779 (55.4)
Previous	61,622 (35.7)	401 (30.9)	30,920 (36.2)	30,301 (35.1)
Current	13,347 (7.7)	65 (5.8)	5,240 (6.1)	8,042 (9.3)
Alcohol, *n* (%)				
None	10,299 (6.0)	149 (13.3)	5,046 (6.0)	5,140 (6.0)
Low	34,665 (20.1)	373 (33.1)	16,906 (19.8)	17,386 (20.2)
Medium	42,789 (24.8)	295 (26.2)	21,622 (25.2)	20,872 (24.2)
High	85,044 (49.1)	309 (27.4)	41,857 (50.0)	42,878 (49.6)
Sleep, *n* (%)				
Short (<6 h)	7,094 (4.1)	70 (6.2)	3,407 (4.0)	3,617 (4.2)
Moderate (6 h ~ 8 h)	154,809 (89.6)	976 (86.4)	76,704 (89.8)	77,129 (89.4)
Long (>8 h)	10,936 (6.3)	83 (7.4)	5,332 (6.2)	5,521 (6.4)
BMI, *n* (%)				
Thin (<18.5)	920 (0.5)	12 (1.1)	488 (0.6)	420 (0.5)
Health (18.5 ~ 24.0)	46,950 (27.1)	343 (30.4)	24,995 (29.3)	21,612 (25.0)
Overweight (24.0 ~ 28.0)	67,130 (38.9)	389 (34.5)	33,347 (39.0)	33,394 (38.7)
Obesity (>28.0)	57,839 (33.5)	385 (34.0)	26,613 (31.1)	30,841 (35.8)

*n*, number; SD, standard deviation. Created with BioRender.com.

Table 2 outlines the hazard ratios (HRs) for nonunion across E-DII categories. In the unadjusted Model 0, participants with anti-inflammatory dietary profiles (lowest E-DII tertile) exhibited a 2.25-fold reduced risk of nonunion (95% CI: 1.47–3.44) compared to those with pro-inflammatory profiles (highest tertile). This association persisted in Model 1 (minimally adjusted; HR: 1.88, 95% CI: 1.13–3.08) and remained significant after adjusting for health-related covariates in Model 2 (HR: 1.87, 95% CI: 1.13–3.08). Further adjustment for lifestyle factors (smoking, alcohol consumption, sleep duration, and educational attainment) in Model 4 continued to demonstrate a robust inverse relationship between anti-inflammatory dietary patterns and nonunion risk. Although no significant differences emerged for the intermediate E-DII tertile, a statistically significant trend (p-trend <0.05) was observed across tertiles (Table 2; Supplementary Tables S1–S4).

**Table 2:** Associations between E-DII and nonunion were investigated by E-DII categories and the continuous score using Cox proportional hazard models.

Nonunion	Pro-inflammatory	Neutral		Anti-inflammatory		Trend	
	HR (95% CI)	HR (95% CI)	*p*-value	HR (95% CI)	*p*-value	HR (95% CI)	*p*-value
Model 0	1.00 (Ref.)	1.01 (0.91; 1.12)	0.83	2.25 (1.47; 3.44)	<0.001	2.23 (1.61; 3.07)	<0.05
Model 1	1.00 (Ref.)	0.93 (0.82; 1.05)	0.22	1.88 (1.13; 3.08)	0.01	2.02 (1.37; 2.93)	<0.05
Model 2	1.00 (Ref.)	0.93 (0.82; 1.05)	0.22	1.87 (1.13; 3.08)	0.01	2.02 (1.37; 2.93)	<0.05
Model 3	1.00 (Ref.)	0.94 (0.83; 1.07)	0.36	1.89 (1.45; 3.11)	0.01	2.01 (1.75; 2.91)	<0.05

Individuals in the pro-inflammatory category were used as the referent. Model 0 only analyzed the correlation between E-DII and nonunion of bones. Model 1 included age, gender, race and economic status. Model 2 newly introduced health-related variables. Model 3 further added lifestyle-related factors.

**Figure 3:**
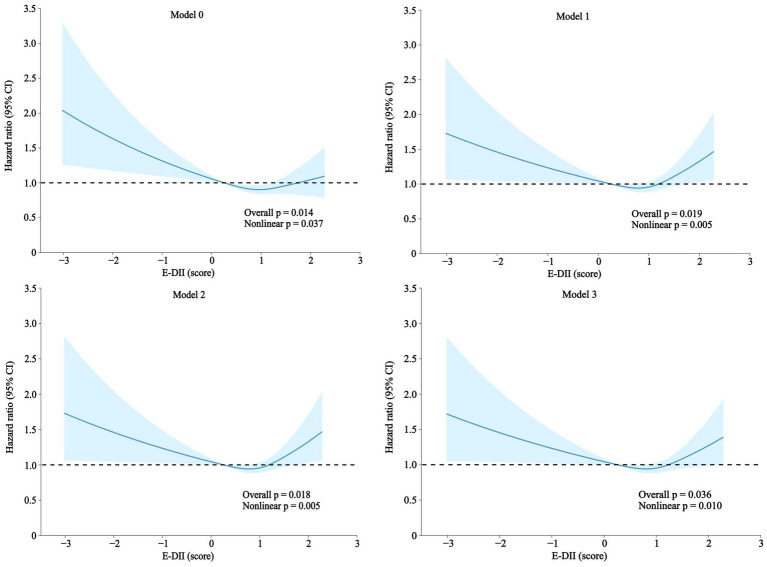
Association between the E-DII and nonunion. A nonlinear association between the E-DII and nonunion was investigated using penalized cubic splines fitted in Cox proportional hazard models. Analyses were performed using the same information reported in Table 2. Created with BioRender.com.

The nonlinear dose–response relationship between cumulative E-DII scores and nonunion risk, illustrated in Figure 3, revealed U-shaped associations across all models (Models 0–3). Both E-DII values below 0.3 and above 1.2 were associated with elevated nonunion risk. Notably, critical thresholds diverged across models: in Model 0 (unadjusted), risk escalation occurred at E-DII > 1.7, whereas Model 1 (minimally adjusted) identified a lower threshold (E-DII > 1.2). Models 2 and 3, incorporating additional confounders, replicated this pattern, with inflection points aligning closely with Model 1.

As shown in the graphical representation, all models demonstrate a U-shaped association between the E-DII score and the risk of nonunion. In Model 0, an E-DII score ranging from 0.3 to 1.7 is linked to a lower risk of nonunion. In Models 1 to 3, an E-DII score within the range of 0.3 to 1.2 is linked to a lower risk of nonunion. Specifically, E-DII scores below 0.3 are associated with a significant increase in the hazard ratio (HR), while scores above 1.2 or 1.7 (depending on the model) show a gradual increase in HR. Overall, the trends across all models are broadly consistent (Table 2). These results highlight the substantial link between dietary inflammatory potential and the risk of nonunion.

**Figure 4:**
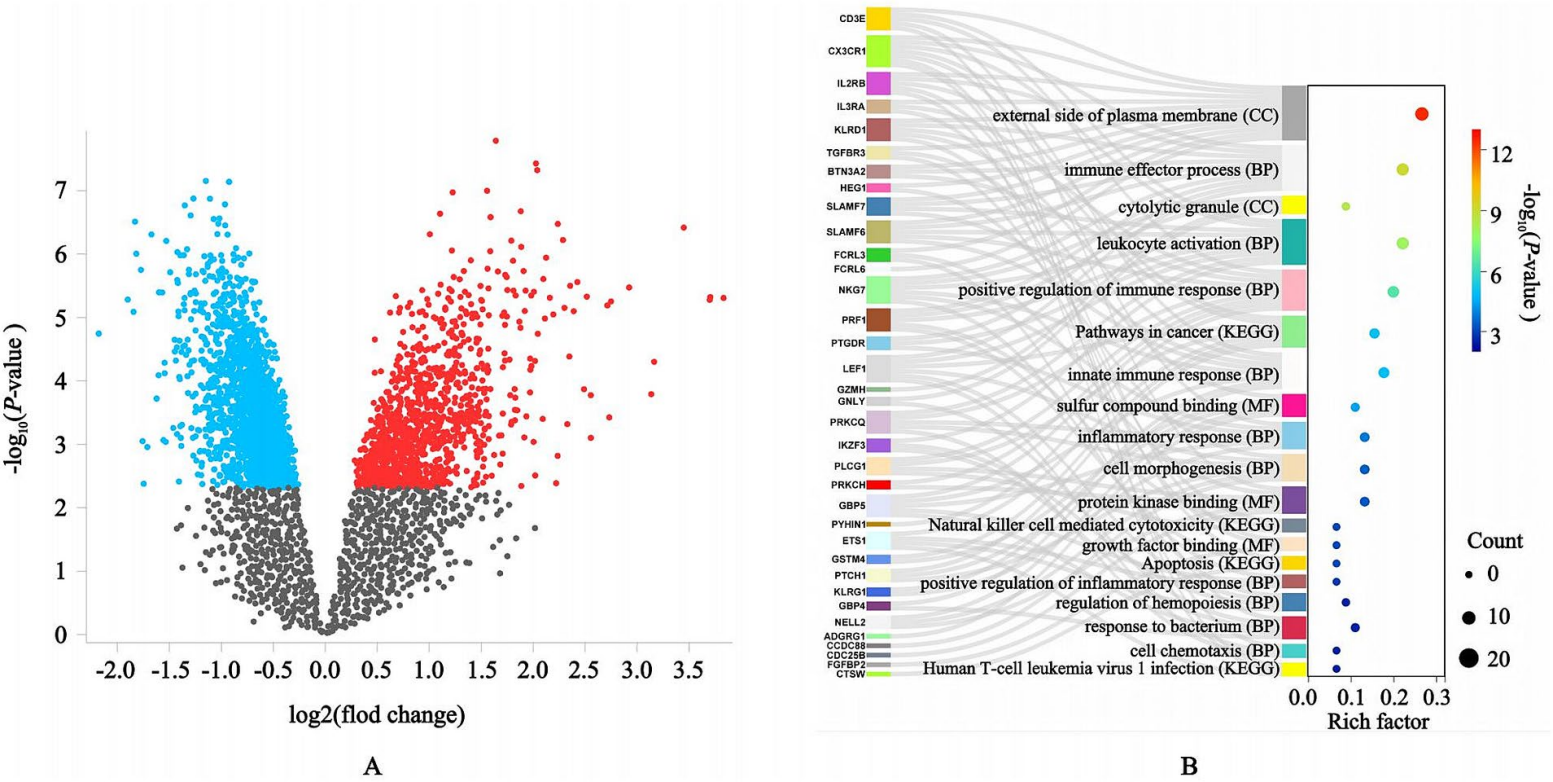
Differential genes and enrichment analysis related to inflammation. **(A)** There were significantly different genes between the patients with acute bone injury nonunion and the healthy volunteers; **(B)** These 35 differential genes were mainly enriched in immune effector processes, leukocyte activation, and the positive regulation of immune responses.

To further investigate the correlation between bone nonunion and inflammatory diets, we identified 44 significantly differentially expressed genes in patients with bone nonunion compared to healthy volunteers (Supplementary Table S5). Among these, 35 genes that were significantly downregulated in inflammation showed interrelated expression patterns (Table 3). Gene enrichment analysis indicated that the 35 differentially expressed genes were mainly associated with immune effector processes, leukocyte activation, and the positive regulation of immune responses (Figure 4B). The complete list of differentially expressed genes in bone nonunion is provided in Supplementary Table S6 and Figure 4A, while the significantly downregulated genes associated with inflammation are detailed in Supplementary Table S7.

**Table 3:** 35 inflammatory genes with significant differences.

Gene symbol	Log FC	adj.P.Val	B	*t*
CTSW	−1.8444463	0.001306	3.986951	−6.9851817
PRF1	−1.8178338	0.000770	5.990396	−8.4541816
TGFBR3	−1.7726300	0.000899	5.440294	−8.0279516
GBP4	−1.6723500	0.000589	6.641569	−8.9846037
IL3RA	−1.6224225	0.006531	0.919465	−5.0842716
IL2RB	−1.5984238	0.001098	4.663977	−7.4569867
FCRL6	−1.5683025	0.001098	4.661159	−7.4549750
ADGRG1	−1.5453712	0.003226	2.194855	−5.8343501
ETS1	−1.5285263	0.000669	6.424688	−8.8046507
PTCH1	−1.4494613	0.002086	3.005067	−6.3386816
KLRG1	−1.4472413	0.002618	2.592242	−6.0786940
GBP5	−1.4367725	0.003110	2.272217	−5.8814839
NKG7	−1.4225088	0.000963	5.091836	−7.7674630
CDC25B	−1.4223500	0.000770	6.046139	−8.4984529
CD3E	−1.4011312	0.002134	2.972240	−6.3177669
PTGDR	−1.3979625	0.004357	1.614685	−5.4871388
PLCG1	−1.3940275	0.000770	5.940647	−8.4148439
GNLY	−1.3939350	0.011619	−0.062916	−4.5352621
NELL2	−1.3893063	0.014923	−0.477587	−4.3091913
KLRD1	−1.3841750	0.017960	−0.764165	−4.1544854
SLAMF6	−1.3531325	0.001113	4.471354	−7.3204054
CCDC88C	−1.3525325	0.001306	4.038906	−7.0205909
HEG1	−1.3507075	0.000475	7.609086	−9.8312904
PYHIN1	−1.3503437	0.002344	2.806969	−6.2131110
IKZF3	−1.3108325	0.000938	5.293127	−7.9170727
FCRL3	−1.3063800	0.003362	2.125795	−5.7924464
CX3CR1	−1.2936813	0.001098	4.64378	−7.4425750
GZMH	−1.2855700	0.003845	1.852789	−5.6283369
PRKCH	−1.2680575	0.000448	7.835947	−10.0410824
SLAMF7	−1.2568300	0.001098	4.726377	−7.5016490
GSTM4	−1.2463200	0.005884	1.094429	−5.1844189
PRKCQ	−1.24369500	0.000938	5.291137	−7.9155819
FGFBP2	−1.2401325	0.025781	−1.284988	−3.8757960
LEF1	−1.2227125	0.012607	−0.194908	−4.4629899
BTN3A2	−1.2079475	0.020549	−0.967741	−4.0452207

## 4 Discussion

In this study, which included 172,839 participants, we observed that individuals with lower E-DII scores were at a higher risk of bone nonunion. The relationship between inflammatory diets and bone nonunion displayed a U-shaped pattern. After adjusting for other covariates, this trend remained significant. Anti-inflammatory diets characterized by deficiencies in essential pro-inflammatory nutrients (e.g., proteins, fatty acids, and carbohydrates) may increase fracture nonunion risk (59). This likely occurs through suppression of the initial inflammatory response required for skeletal healing. Specifically, inadequate inflammation impairs immune cell recruitment to the fracture site, reduces necrotic tissue clearance, diminishes growth factor release, and disrupts stem cell homing and angiogenesis. These deficits collectively delay or compromise callus formation, elevating nonunion susceptibility (60). Conversely, hyper-proinflammatory diets rich in refined carbohydrates, excessive calories, and saturated fats promote adipose tissue inflammation, gut dysbiosis, and endotoxemia (61). This induces a persistent systemic low-grade inflammatory state that amplifies and prolongs the local inflammatory response at the fracture site. The resulting chronic inflammation impedes resolution of the healing cascade, ultimately contributing to nonunion (62). Notably, the nadir of the U-shaped E-DII risk curve resides within a mildly proinflammatory range. This strategic positioning permits adequate initiation of the essential inflammatory phase while providing sufficient anti-inflammatory and pro-repair nutrients to facilitate timely inflammation resolution after initial healing tasks are completed.

Given the increasing diversity in people’s diets and the varied channels for obtaining food, leading to an unbalanced dietary structure, coupled with the rising incidence of nonunion, there is an urgent need to explore deeper pathogenic factors to facilitate early prevention. Therefore, the impact of dietary patterns on disease has become an important area of research, attracting high social attention.

When elucidating the pathophysiology of bone non-union, chronic low-grade inflammation is increasingly regarded as a pivotal contributor. Accordingly, our findings should be interpreted against the backdrop of mounting evidence that dietary patterns can modulate bone healing by altering systemic inflammatory status. A substantial body of work has demonstrated that specific dietary choices exert a pronounced influence on circulating inflammatory biomarkers (63, 64). Among these biomarkers, CRP is a highly sensitive indicator of systemic inflammation, and fluctuations in its concentration provide a rapid read-out of the body’s inflammatory status (65). Notably, dietary patterns enriched in anti-inflammatory constituents—such as specific fruits and vegetables (66), n-3 polyunsaturated fatty acids (67, 68), and dietary fiber (69)—have been consistently associated with markedly lower circulating CRP concentrations, whereas pro-inflammatory components, exemplified by certain refined carbohydrates (70), appear to exert the opposite effect. To quantify the overall inflammatory potential of an individual’s diet, analytical tools such as the DII and its E-DII have been developed (24, 29, 71, 72).

Multiple investigations conducted across Europe and North America have consistently shown that higher E-DII scores—reflecting more pro-inflammatory dietary patterns—are significantly associated not only with elevated CRP concentrations but also with increased levels of other pivotal pro-inflammatory cytokines, including interleukin-6 IL-6 and TNF-*α* (73, 74). Given that CRP, IL-6, TNF-α and related inflammatory mediators have been shown to disrupt essential phases of fracture repair—including osteoblast differentiation, angiogenesis and extracellular-matrix deposition—and to impede callus formation, a sustained, diet-driven low-grade inflammatory milieu is very likely a key modifiable factor that heightens the risk of bone non-union. Accordingly, the association we observed between a pro-inflammatory dietary profile and non-union risk is most plausibly mediated by diet-regulated inflammatory biomarkers and their downstream effects on the local bone-healing microenvironment.

Following skeletal fracture, a coordinated cascade of molecular and cellular events is initiated at the injury site. The initial inflammatory stage of bone healing is characterized by the rapid recruitment of a diverse array of immune cells and pro-inflammatory mediators, culminating in hematoma formation. This provisional matrix serves as a biological scaffold essential for orchestrating subsequent reparative mechanisms (75). Systemic inflammatory mediators—including CRP, IL-6 and TNF-*α*—are produced chiefly by activated macrophages, as well as by neutrophils, lymphocytes and other cells of the innate and adaptive immune systems (76). Any factor that alters immune-cell activation, polarisation or recruitment can therefore reshape the circulating cytokine milieu. Dietary modifications, pharmacological agents and lifestyle interventions that attenuate pro-inflammatory macrophage activity or foster anti-inflammatory immune phenotypes represent indirect yet effective strategies for regulating serum levels of these biomarkers.

Animal experiments have demonstrated that the absence or delayed infiltration of macrophages in the early stages of fracture significantly increases the risk of nonunion (77–79). Further research on macrophages has similarly shown that these cells play a crucial role in fracture healing by secreting large amounts of prostacyclin, which promotes angiogenesis and modulates the activity of osteoblasts and osteoclasts, thereby enhancing fracture repair (80, 81). Cyclooxygenase (COX) inhibitors, both selective and non-selective, are commonly used as analgesics in clinical practice to effectively inhibit COX production. However, animal studies have shown that COX-2 deficient mice exhibit severe nonunion (82, 83), while the administration of prostacyclin agonists can reverse this condition (84). By blocking the conversion of arachidonic acid to prostaglandins, COX inhibitors diminish macrophage activation, suppress the sustained release of pro-inflammatory mediators such as IL-6 and TNF-*α*, and lower circulating CRP (85). This attenuation of the inflammatory cascade not only relieves pain and exudation but also creates a microenvironment more conducive to tissue repair. Our studies, together with prior animal investigations, indicate that excessive—whether direct or indirect—suppression of circulating inflammatory mediators can exacerbate bone non-union. Consequently, the prolonged use of cyclo-oxygenase inhibitors in patients with established non-union warrants careful re-evaluation and constitutes a critical avenue for future orthopaedic research.

Restricted cubic spline analysis indicated that the lowest predicted probability of non-union occurred at an E-DII value of approximately 1. Deviations in either direction—towards more pro- or anti-inflammatory dietary profiles—were associated with higher non-union risk, with a steeper gradient on the anti-inflammatory side. Because these findings arise from an observational design, they should not be interpreted as evidence of causality; rather, they suggest a non-linear relationship in which an intermediate dietary inflammatory potential may coincide with more favourable fracture healing. Previous studies have shown that mild inflammatory initiation seems to accelerate repair; As the research has proved, the healing time of subsequent fractures is approximately half that of the first fracture. Further studies have shown that secondary injury after fracture can stimulate fracture healing. For instance, a clinical study by Borden et al. (84, 85) reported that the nonunion rate of femoral neck fractures fixed within 6 days after injury was 25%, while the nonunion rate of surgeries delayed by more than 7 days was only 7% (86, 87). The authors of the study believe that a short delay allows for limited micro-movement at the fracture site, promotes the accumulation of local inflammatory mediators, and creates a mild inflammatory environment conducive to callus formation and bone repair.

A mild increase in the levels of inflammatory factors in the body can promote fracture healing and reduce the probability of fracture nonunion, while excessive increase or decrease in the levels of inflammatory factors are both important factors increasing the risk of bone nonunion. In recent years, relative research has demonstrated that excessive inflammatory stimulation actually inhibits the recruitment of macrophages to the fracture site, thereby increasing the risk of nonunion (88). The activation of inflammatory factors in the early stage can promote fracture healing, but when inflammation turns into chronic inflammation, it will inhibit fracture healing and increase the risk of nonunion (62). A number of studies have shown that immunodeficiency can lead to severe impairment of fracture healing (89, 90), or the lack of macrophages at the fracture site may also cause nonunion (91, 92). Multiple studies concur that a modest rise in inflammatory mediator levels can enhance fracture healing, whereas both excessive inflammation and marked suppression of these mediators are linked to a higher incidence of bone non-union.

Recent evidence indicates that diet-induced obesity can predispose to bone non-union by altering neutrophil function; mechanistic studies further implicate activation of the NLRP3 inflammasome as the critical intermediary in this process (41). Because a mildly pro-inflammatory diet can raise circulating cytokine levels to a range that supports fracture repair, we next investigated the gene targets through which a strongly anti-inflammatory dietary pattern might predispose to bone non-union (93). To explore the paradoxical association between anti-inflammatory dietary patterns and elevated bone nonunion risk, we interrogated transcriptomic profiles of nonunion patients sourced from the Gene Expression Omnibus (GEO) database. Comparative analysis identified 35 inflammation-associated genes exhibiting significant downregulation in nonunion cases relative to controls. Notably, critical mediators of inflammatory regulation—including CD3E, CX3CR1, IL2RB, and IF3RE—were suppressed in nonunion tissues. To systematically identify bone-non-union gene targets that may be modulated by an anti-inflammatory diet, we integrated multiple publicly available transcriptomic datasets generated on diverse sequencing platforms and from varied sources. Batch-effect correction and cross-validation were applied to maximise the robustness and reliability of the findings. These findings suggest that attenuated expression of inflammatory genes may underlie compromised fracture healing in individuals adhering to anti-inflammatory diets, though mechanistic drivers require further elucidation.

## 5 Strength and limitations

The UK Biobank, as a large-scale and highly integrated database, provides an essential platform for investigating issues relevant to the general population. This study utilized data from the UK Biobank to examine the association between nonunion of bones and inflammatory diet. Given that both exposure and outcome data were sourced from the UKB database, the potential for information bias was significantly minimized. Additionally, this study incorporated extensive data on confounding factors. By adjusting for these confounders, we assessed the correlation between nonunion of bones and inflammatory diet, as well as the consistency of trends across different models.

Using the UK Biobank as our research platform is a significant advantage of our study, but it also has several limitations. Firstly, depending on a single database restricts the generalizability of our results to the wider human population and might not adequately reflect all global demographic groups. Therefore, in necessary circumstances, we will verify whether the results of this study are universal in multiple databases. Secondly, although we included numerous confounding factors in our analysis, unmeasured or residual confounding remains a possibility. Third, the E-DII scores were calculated based on self-reported dietary information, which is vulnerable to recall bias and possible misclassification of food categories. Finally, the datasets employed for target gene screening in this study capture associations between inflammatory markers and fracture nonunion; they do not directly reflect correlations between E-DII and nonunion.

Therefore, the findings of this study should not be interpreted as establishing a causal link between inflammatory diets and bone nonunion. This research can only indicate a significant association between inflammatory diets and bone nonunion; however, it is not feasible to use DII or E-DII scores to determine the prevalence or incidence of bone nonunion (94, 95).

## 6 Conclusion

In sum, we observed a statistically significant, nonunion relationship between E-DII values and the probability of nonunion: both markedly pro- and anti-inflammatory scores were linked to higher risk, whereas a mildly pro-inflammatory range corresponded to the lowest risk. Although these data highlight diet as a potentially modifiable factor in fracture healing, the observational design limits causal interpretation. Future research should therefore prioritise prospective cohorts and controlled dietary interventions to clarify causality, quantify the impact of targeted nutritional modifications, and identify complementary lifestyle or clinical measures that might reduce the burden of nonunion. Particular emphasis on the complex effects of strongly anti-inflammatory diets is warranted to inform evidence-based management strategies.

## Data Availability

The raw data supporting the conclusions of this article will be made available by the authors, without undue reservation.

## Footnotes

^1^ http://www.ukbiobank.ac.uk
